# Downregulation of Fat Mass and Obesity-Related Protein in the Anterior Cingulate Cortex Participates in Anxiety- and Depression-Like Behaviors Induced by Neuropathic Pain

**DOI:** 10.3389/fncel.2022.884296

**Published:** 2022-05-12

**Authors:** Xiao-Ling Wang, Xin Wei, Jing-Jing Yuan, Yuan-Yuan Mao, Zhong-Yu Wang, Na Xing, Han-Wen Gu, Cai-Hong Lin, Wen-Ting Wang, Wei Zhang, Fei Xing

**Affiliations:** ^1^Department of Anesthesiology, Pain and Perioperative Medicine, The First Affiliated Hospital of Zhengzhou University, Zhengzhou, China; ^2^Henan Province International Joint Laboratory of Pain, Cognition and Emotion, Zhengzhou, China; ^3^Department of Human Anatomy, School of Medical Sciences, Zhengzhou University, Zhengzhou, China; ^4^Department of Anesthesiology and Perioperative Medicine, The Second Affiliated Hospital of Zhengzhou University, Zhengzhou, China

**Keywords:** neuropathic pain, anterior cingulate cortex, anxiety-like behavior, depression-like behavior, fat mass and obesity-related protein, matrix metalloproteinase-9

## Abstract

N6-methyladenosine (m^6^A) is the most abundant methylation modification on mRNA in mammals. Fat mass and obesity-related protein (FTO) is the main RNA m^6^A demethylase. FTO is involved in the occurrence and maintenance of neuropathic pain (NP). NP often induces mental disorders. We found that NP downregulated the expression of FTO in the anterior cingulate cortex (ACC), inhibited the expression of matrix metalloproteinase-9 (MMP-9) in the ACC, maladjusted the brain-derived neurotrophic factor precursor (proBDNF) and mature brain-derived neurotrophic factor (mBDNF) levels in the ACC, and induced anxiety- and depression-like behaviors in mice. Blocking the downregulation of FTO in the ACC induced by peripheral nerve injury could reverse the anxiety- and depression-like behaviors of mice. Contrarily, downregulation of simulated FTO induced anxiety- and depression-like behaviors in mice. After peripheral nerve injury, the binding of FTO to MMP-9 mRNA decreased and the enrichment of m^6^A on MMP-9 mRNA increased. In conclusion, downregulation of FTO in ACC by regulating MMP-9 mRNA methylation level contributes to the occurrence of anxiety- and depression-like behaviors in NP mice.

## Introduction

Chronic pain usually refers to continuous or intermittent pain of more than three months. It is an unpleasant feeling and emotional experience. Neuropathic pain (NP) caused by somatic nervous system diseases and other diseases is a common chronic pain characterized by central or peripheral nervous system damage and refractory and unknown mechanisms. Clinical research shows that chronic pain, as a stress state, often induces depression and is one of the primary determinants of emotional disorders (von Knorring et al., 1983; Lee et al., 2009; Aguera-Ortiz et al., 2011). Chronic pain is one of the primary determinants of emotional disorders. Up to 54% and 50% of patients with chronic pain suffer from depression and anxiety, respectively (Fishbain et al., 1997; Bair et al., 2003; Williams et al., 2003). One clinical study showed that, the incidence of mood disorders was 47% in NP patients’ lifetime (Radat et al., 2013). According to previous studies, the prognosis of patients with chronic pain-associated depression is worse than that of patients with simple chronic pain. Chronic pain and depression are closely related in occurrence and development, promote each other, and accelerate the development of each other’s severity (Fishbain et al., 1997). Contrarily, the underlying basis of this comorbidity and the dynamic interaction between pain and depression remain unclear.

In recent years, RNA methylation-mediated RNA posttranscriptional modification has attracted much attention as an important epigenetic modification. N6-methyladenosine (m^6^A) is one of the most common RNA modifications; it is found in >25% of mammalian mRNA (Dominissini et al., 2012; Cui et al., 2016). Studies have shown that fat mass and obesity-related protein (FTO) can participate in RNA posttranscriptional modification as a demethylase. It plays a significant role in various biological advances by regulating mRNA metabolism, including RNA splicing. After peripheral nerve injury, the expression of FTO in the dorsal root ganglion (DRG) increased, whereas the level of m^6^A regulated by FTO decreased. Blocking this increase can reverse m^6^A loss caused by nerve injury and reduce nerve injury-related hyperalgesia, suggesting that FTO participates in the peripheral mechanism of NP in an RNA m^6^A-dependent manner (Li et al., 2020). According to a previous study, FTO, which was found to be highly expressed in brain tissues, participated in Alzheimer’s disease (AD) through its effect on mTOR in AD mouse models (Li et al., 2018). Knockdown of FTO can reverse tau protein hyperphosphorylation level and improve the cognitive defect of Aβ aggregation in AD mouse models (Van Skike et al., 2018). In addition, chronic restraint stress can downregulate the expression of FTO in mouse hippocampus, and thus induce anxiety- and depression-like behaviors in mice (Shen et al., 2021). However, whether FTO in ACC is involved in the occurrence or maintenance of anxiety- and depression-like behaviors caused by NP remains unclear.

Matrix metalloproteinases (MMPs) play an important role in the structural development and maintenance of the nervous system. The MMP family includes more than 20 members; one member, MMP-9, is involved in angiogenesis, axon growth, and neural plasticity (Cañete Soler et al., 1995; Jaworski and Fager, 2000; Hayashita-Kinoh et al., 2001). Accumulating evidence show that MMPs play an important role in the occurrence of NP (Jiang et al., 2019). Several studies have shown that the expression of the MMP-9 protein decreased in the hippocampus of FTO knockout mice, and MMP-9 participated in the regulation of anxiety- and depression-like behaviors in mice by affecting the transformation of brain-derived neurotrophic factor precursor (proBDNF) to mature brain-derived neurotrophic factor (mBDNF). As a brain region shared by the physical pain sensory pathway and emotion management, ACC constitutes the histological basis for the coexistence of pain and emotional disorders. This coexistence is critical for the occurrence and development of chronic pain-induced depression. However, whether MMP-9 in ACC is involved in the occurrence or maintenance of anxiety- and depression-like behaviors caused by NP remains unclear.

In this study, the expression of FTO was downregulated in ACC of NP mice -induced by chronic constriction injury. Therefore, we hypothesized that downregulated FTO in ACC contributes to anxiety- and depression-like behaviors-induced by NP by increasing m^6^A level of MMP-9 mRNA. The study sought to indicate that FTO regulates the participation of MMP-9 in the generation and maintenance of anxiety and depression-like behaviors under NP conditions.

## Materials and Methods

### Animals

Male C57BL/6J mice weighing 20–30 g (6–8 weeks old, used for *in vivo* experiments) were purchased from the Laboratory Animal Center of Zhengzhou University, Zhengzhou, China. They were raised intensively at the School of Basic Medicine of Zhengzhou University. The room temperature was maintained at 23 ± 2°C under a natural light–dark cycle. All animal experimental procedures were approved by the Institutional Animal Care and Use Committee of Zhengzhou University; they were performed according to the guidelines of the National Institutes of Health (NIH) on animal care.

### Animal Model

The CCI model was used, as previously described (Bennett and Xie, 1988). Simply, the animals were anesthetized with 1.5–2% isoflurane/oxygen. The sciatic nerve of the left leg was bluntly separated and loosely ligated with three 5-0 catgut wires (Busan Ailee Co., Ltd., South Korea), with about 1 mm. The skin and muscles were sutured with non-absorbable 5-0 silk thread. The Sham operation group underwent the same operation, except that the sciatic nerve was not ligated. After the operation, the mice were sent back to the cage for recovery and free access to food and water.

### Stereotaxic Surgery

The animals were anesthetized with 1.5–2% isoflurane/oxygen and placed in a stereotactic frame. The scalp was incised, and skull holes were drilled. The viral vector (300 nl, per side, 30 nl/min) was microinjected into the right ACC (coordinates, AP + 1 mm, ML + 0.3 mm, DV - 1.4 mm). Microtubules were kept *in situ* for 10 min after microinjection to ensure carrier diffusion. Thereafter, the microtubules were slowly pulled out, and the incisions were closed.

### Behavioral Tests

Behavioral tests included pain behavioral test and anxiety- and depression-like emotional behavioral test. Pain behavior test mice were taken to the test room 30 min before each test. All mice adapted to the test environment at least 3 days before baseline measurement. Mice undergoing anxiety- and depression-like behavioral tests were brought into the test room 24 h prior to the tests. Anxiety- and depression-like behavioral tests included open field test (OFT), elevated plus maze (EPM), tail suspension test (TST), and forced swimming test (FST). The trajectories of mice were recorded using a computer tracking system (smart 3.0, Panlab Harvard, Holliston, MA, United States). All the evaluators were blinded to the experimental design.

#### Paw Withdrawal Threshold

Paw withdrawal threshold was detected by assessing 50% withdrawal thresholds to evaluate mechanical sensitivity using a set of von Frey filaments (0.02–4 g, Stoelting, Wood Dale, IL, United States), following an up-down method, as previously described. PWT was assessed a day prior to CCI and 3, 7, 14, 21, and 28 days after CCI (Chaplan et al., 1994). Mice were placed in a cage with a wire mesh bottom allowing full access to the paws. The paws were touched with a series of von Frey filaments in ascending order of strength, at intervals allowing for resolution of behavioral response to the previous stimuli. Sharp paw withdrawal, paw licking, and flinching were interpreted as positive responses.

#### Paw Withdrawal Latency

The evaluation of heat hypersensitivity was assessed by PWL using a plantar analgesia tester (7370, Ugo Basile, Comerio, Italy), according to the protocols published by Hargreaves et al. (1988). Briefly, the radiant heat source placed under the glass was aimed at the plantar surface of the hind paw. The latency was measured thrice for each hind paw in every test session. The hind paw was alternately analyzed with an interval of >5 min between the consecutive detections. The average of the three measurements of latency per side was used as the final result. All the evaluators were blinded to the experimental design.

#### Open Field Test

The OFT is a classic test of general locomotor activity in rodents; it was performed in this study, as previously described (Ferreira-Chamorro et al., 2018). Each mouse was placed in the center of an open arena (40 × 40 × 40 cm^3^) and allowed to freely explore the arena for 5 min. The time spent in the central area, number of times, and total distance covered in the central area were recorded.

#### Elevated Plus Maze

Elevated plus maze is the most used rodent model of anxiety (Kobayashi et al., 2008). It is based on rodents’ natural aversion to open spaces and leads to a behavior, which involves avoidance of open areas by confining movements to enclosed spaces. The EPM was situated in a separate brightly lit room that was illuminated with overhead lights, which provided a light density of approximately 300 lx at the center and the arms of the EPM. The EPM consisted of a cross-shaped platform (height: 60 cm) with four arms (width: 6 cm; length: 40cm), two of which were enclosed by walls (height: 30 cm). The mice from each group were placed in the center of this cross facing an open arm and were free to explore the EPM for 5 min. Their activities were recorded. Anxiety was indicated by an increase in the proportion of time spent in the closed arms and an increase in the proportion of entries into the closed arms.

#### Tail Suspension Test

To test depression-like behavior, TST was performed, as previously described (Steru et al., 1985). Briefly, mice were suspended using adhesive tapes wrapped around their tails 1 cm from the tip and tied to a hook in the observation chamber. Each mouse was suspended for 5 min, and the duration of total immobility was measured.

#### Forced Swim Test

Briefly, Mice were individually placed in glass cylinders (diameter and depth 10 × 25 cm) filled with water (25 ± 1°C) up to 19 cm. Mice were forced to swim for 6 min, and the immobility time during the last 5 min was manually measured by a blinded observer. Mice were considered immobile when they ceased struggling, remained floating motionless, and only made those movements necessary to keep their head above the water.

### Western Blot

Mice were killed under anesthesia with 5% chloral hydrate (300 mg/kg), which was injected intraperitoneally. The ACC were harvested on ice and lysed in ice-cold radioimmunoprecipitation assay (RIPA) lysis buffer (Applygen Technologies Inc., Beijing, China), which contains protease inhibitors. Homogenates were centrifuged at 12,000 rpm for 10 min at 4°C, and the protein concentrations were determined using the bicinchoninic acid (BCA) method. Thereafter, 8% sodium dodecyl sulphate-polyacrylamide gel electrophoresis (SDS-PAGE) was applied to separate protein samples, which were then transferred to a polyvinylidene difluoride (PVDF) membrane with 200 mA current for 1.5 h. Subsequently, the membranes were blocked using 3% non-fat milk for 1 h at room temperature and incubated with specific primary antibodies, including FTO (1:1000, Abcam, ab92821), MMP-9 (1:1000, ab58803, Abcam, United Kingdom), proBDNF (1:1000, sc-65514, Santa), mBDNF (1:1000, ab108319, Abcam, United Kingdom), histone-h3 (H3) (1:5000, ab1791, Abcam, United Kingdom), and glyceraldehyde 3-phosphate dehydrogenase (GAPDH) (1:2,000, ab9485, Abcam, United Kingdom) at 4°C overnight. After being washed thrice, the membranes were incubated in a goat anti-rabbit immunoglobulin G (IgG) horseradish peroxidase (HRP)-linked antibody (1:2,000, ab6721, Abcam, United Kingdom) for 2 h at room temperature. Thereafter, they were incubated with sheep anti-mouse or anti-rabbit secondary antibody at room temperature for 2 h. The chemiluminescence reagent was detected with the ECL Kit (bio-RAD) on a Protein Simple machine (Fluorchem, United States). A computer-aided image analysis system (ImageJ, NIH, United States) was used to quantify the imprint intensity. Each experiment was repeated at least thrice. The imprinting density of the control group was 100%. The values of other groups were normalized by the optical density values of these groups and the control group to the relevant GAPDH or H3 optical density.

### RNA Extraction and Quantitative Real Time Polymerase Chain Reaction

Total RNA was extracted from samples using RNA simple total RNA Extraction Kit (TIANGEN, DP419). For reverse transcription and obtaining cDNA, Revert Aid First Strand cDNA Synthesis Kit (Thermo Fisher Scientific, United States) was used. To determine the level of mRNA target genes, thermocycler CFX96 Real-Time PCR Detection Systems (Eppendorf, Germany) and a set of reagents, Maxima SYBR Green/ROX qPCR Master Mix (2X) (Thermo Scientific, United States), were used. Specific primer pairs (5′-3′) for analysis of target and reference genes were selected by the software Primer Blast^1^ and produced by Thermo Fisher Scientific (United States). The sequence of relevant target genes is shown in Table 1. Normalized relative quantity of cDNA target genes was determined using the ^ΔΔ^C_t_ method.

**TABLE 1:** Sequences of primers used.

Gene	Primer	Sequence
FTO	Forward	GCGGGAAGCTAAGAAACTGA
FTO	Reverse	ATGCAGCTCCTCTGGTATGC
MMP-9	Forward	AAAGGCCATTCGAACACCAC
MMP-9	Reverse	GGATGACAATGTCCGCTTCG
GAPDH	Forward	TCGGTGTGAACGGATTTGGC
GAPDH	Reverse	TCCCATTCTCGGCCTTGACT

### Recombinant Adeno-Associated Virus 2/9

Overexpression FTO was designed by Brain VTA (Wuhan) Co., Ltd. Both AAV-FTO and AAV-scramble were sub-cloned into a dual expression adeno-associated virus vector 2/9 (AAV2/9), with CMV driving EGFP expression and HA driving expression of FTO. The titer of these AAV was 1.00E + 12 infectious vg/ml.

Pre-validated shRNA-FTO was obtained from Sigma-Aldrich (TRCN0000277193, sequence: 5′- GTCTCGTTGAAAT CCTTTGAT-3′), and the scrambled shRNA (shRNA-scramble) was a gift from Brain VTA (Wuhan) Co., Ltd. Both shRNA-FTO and shRNA-scramble were sub-cloned into a dual expression adeno-associated virus vector 2/9 (AAV2/9), with CMV driving EGFP expression and HA driving expression of shRNA. The titer of these AAV was ≥ 5.00E + 12 infectious vg/ml.

### RNA Immunoprecipitation Assay

RNA binding protein immunoprecipitation kit (Millipore, Darmstadt, Germany) was used for RNA immunoprecipitation (RIP). ACC tissues were separated into single cell suspensions in ice cold PBS with Dounce homogenizer, and centrifuged at 1500 rpm at 4°C for 5 min. The cells were collected and added to the lysate containing RNase inhibitor and protease inhibitor. RIP lysate was incubated on ice for 5 min and stored at –80°C. The magnetic beads were gently turned upside-down and evenly mixed to make a suspension. Afterward, 500 μl RIP Washing buffer was taken off the magnetic bead sealing solution with L rip washing buffer; 200 μl of RIP washing buffer was added. Thereafter, 5 μl was added to the tube. A/G target protein m^6^A antibody (SYSY, 202111), FTO antibody (Santa, sc-271713), or normal mouse IgG were washed thrice with RIP washing buffer, and the beans protein A/G antibody complex was resuspended in RIP immunoprecipitation buffer. After thawing, the cell lysate was rotated at 14,000 rpm for 10 min at 4°C. The supernatant obtained by centrifugation was added into the magnetic bead antibody RIP precipitation buffer, and the mixture of antibody and bead was incubated overnight at room temperature on a rotary mixer. Thereafter, 1 ml RIP washing buffer was added; the mixture was gently rotated and mixed. Washing was repeated for six times. The beads were incubated in protease K buffer. After shaking at 55°C for 30 min, the RNA was eluted and purified by phenol/chloroform extraction. The enrichment of RNA was detected by real-time fluorescence quantitative PCR. PCR primers are shown in Table 1. The MMP-9 primers is located on the 2 exon of MMP-9.

### Immunofluorescence

Mice were perfused with 4% paraformaldehyde. The brain tissues of mice were dissected quickly, fixed with 4% paraformaldehyde overnight, dehydrated with 30% sucrose overnight, crosscut, and continuously cut into 20 um sections. The sections were kept at room temperature for 1 h, washed with PBS, incubated with mouse FTO antibody, kept at room temperature for another 1 h, rinsed with PBS, and incubated with mouse FTO antibody. They were mixed with NeuN, GFAP, Iba1, or MMP-9 antibody overnight or 48 h at 4°C. After PBS washing, the slices were incubated with fluorescent-labeled secondary antibody, Alexa Fluor 488 goat anti-mouse IgG or Alexa Fluor 546 goat anti-rabbit IgG, at room temperature for 2 h. After PBS washing, DAPI was incubated at room temperature for 10 min and then washed with PBS. Finally, the slices were placed on the slide and covered with the cover slide. The examination was performed under a fluorescence microscope (Olympus bx53, Tokyo, Japan) or a high-resolution laser confocal fluorescence microscope (Nikon A1R MP + Japan), and the images were taken with a CCD spot camera.

### *In situ* Hybridization

*In situ* hybridization was performed, as previously described (Wu and Oh, 1996). MMP-9 *in situ* hybridization detection kit (Boster, mk3897-m) was used for the experiment. Mouse brain tissues were fixed overnight with 4% paraformaldehyde, dehydrated with 20% and 30% sucrose, embedded with OCT, and continuously cut into 20 um sections using a frozen slicer. Thereafter, 4% paraformaldehyde/0.1M PBS (pH: 7.2–7.6) was fixed at room temperature for 10 min. Each slice was incubated with 20 ul prehybridizing solution at 38–42°C for 3 h. Each slice was incubated with 20 ul hybridization solution at 38–42°C overnight. Sealing solution was added dropwise at 37°C for 30 min. Biotinylated rat anti-digoxin was added dropwise at 37°C for 60 min. Additionally, SABC-FITC was added dropwise (1ul SABC-FITC plus PBS 1,001 ul for *in situ* hybridization, 50 ul per slice, 37°C for 30 min). The film was sealed with anti-fluorescence quencher and observed under a high-resolution laser confocal fluorescence microscope (Nikon A1R MP + Japan). The sequence of MMP-9 target gene is: 5—GGGAC CATCA TAACA TCACA TACTG GATCC AAAAC TACTC—3; 5—GGTAC TGGAA GTTCC TGAAT CATAG AGGAA GCCCA TTACA—3; 5—TCTGC CATGG CAAAT TCTTC TGGCG TGTGA GTTTC CAAAA—3.

### Statistical Analysis

Mean ± SEM was used to normally distributed data, which were analyzed with GraphPad Prism 8.0 (GraphPad, San Diego, CA, United States). The data were analyzed using two-tailed, unpaired *t*-Test (two groups) and one-way ANOVA (> 2 groups). For the results on pain behavior, two-way ANOVA was used for data analysis. When the analysis of variance showed significant differences, Tukey’s *post hoc* method was used for pairwise comparison of the mean. The detailed analysis process used in each experiment is described in detail in the legend of the result diagram. *P* < 0.05 was considered as statistically significant.

## Results

### Peripheral Nerve Injury-Induced Chronic Hyperalgesia and Anxiety- and Depression-Like Behaviors in Mice

**FIGURE 1:**
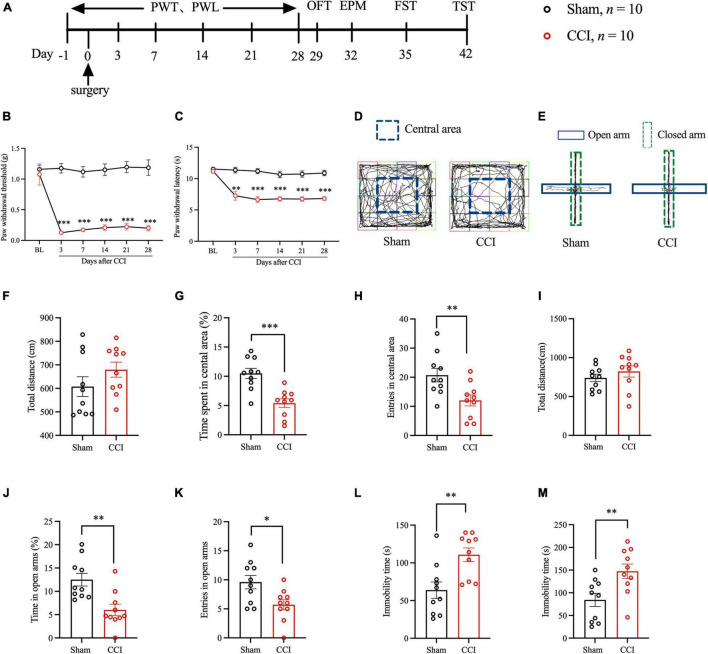
Peripheral nerve injury induced hyperalgesia and anxiety- and depression-like behaviors in mice. **(A)** The schematic diagram of the experimental arrangement. **(B,C)** The paw withdrawal threshold and paw withdrawal latency of mice at -1, 3, 7, 14, 21, and 28 days after CCI (*n* = 10, **P < 0.01 vs. Sham, ***P < 0.001 vs. Sham); **(D)** OFT trajectory. **(F)** The total movement distance of mice was observed during the OFT. **(G)** The proportion of residence time in the central area (*n* = 10, ***P < 0.001 vs. Sham). **(H)** The number of entries of the central area (*n* = 10, **P < 0.01 vs. Sham); **(E)** EPM trajectory. **(I)** The total movement distance of mice during the EPM. **(J)** The proportion of residence time in the open arm (*n* = 10, **P < 0.01 vs. Sham). **(K)** The number of entries of the open arm (*n* = 10, *P < 0.05 vs. Sham); **(L,M)** The immobility time during the FST and TST (*n* = 10, **P < 0.01 vs. Sham).

We evaluated hyperalgesia in mice after CCI by PWT and PWL at -1, 3, 7, 14, 21, and 28 days after surgery (Figure 1A). The pain behavioral tests showed that the PWT and PWL values of CCI model mice decreased significantly (Figures 1B,C), suggesting that the NP mouse model was successfully prepared, and this finding was consistent with that of the previous studies. Thereafter, we examined whether NP induced anxiety- and depression-like behaviors with several behavioral tests. The results of the OFT showed that, compared with the Sham group, the CCI group had a significantly reduced percentage of time spent in the central area and entries of the central area after the surgery. However, no significant difference in total movement distance was detected (Figures 1D,F–H). The EPM results showed that, compared with the Sham group, the CCI group had a significantly decreased percentage of time in the open arms and entries of the open arms were significantly descended after surgery. However, no significant difference in the total movement distance was recorded (Figures 1E,I–K). Regarding FST and TST, the CCI group had a more prolonged time of immobility after surgery than the Sham group (Figures 1L,M). These results suggested that NP could lead to anxiety- and depression-like behaviors in mice.

### Neuropathic Pain Decreased the Expression of Fat Mass and Obesity-Related Protein in Anterior Cingulate Cortex

**FIGURE 2:**
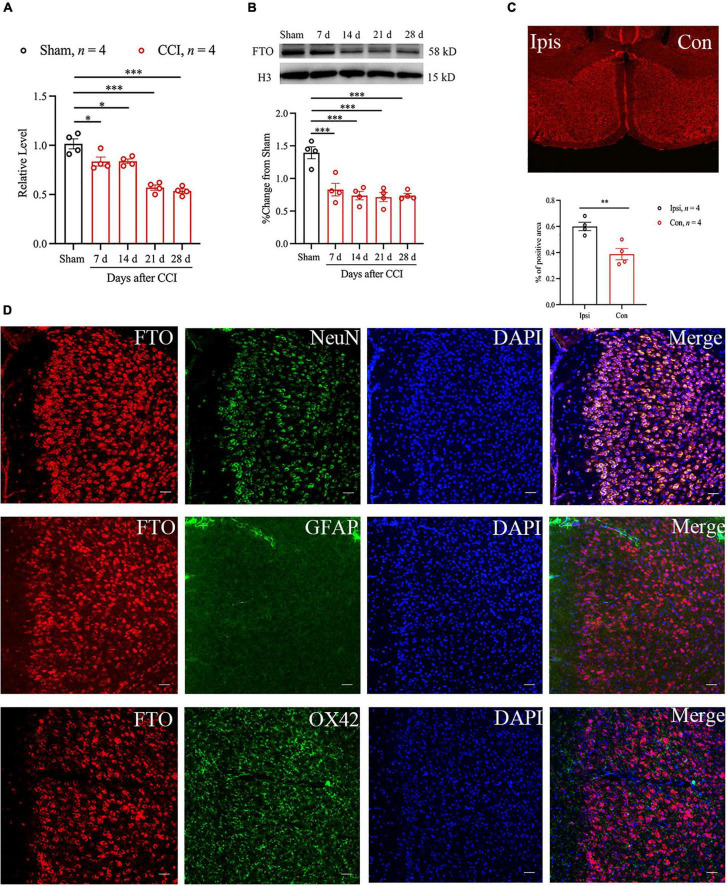
Peripheral nerve injury can reduce the expression of FTO protein and FTO mRNA in mice ACC. **(A,B)** qRT-PCR and Western blot results showed that the levels of FTO mRNA and protein in ACC of mice decreased significantly 7, 14, 21, and 28 days after CCI (*n* = 4, *P < 0.05, ***P < 0.001 vs. Sham). **(C)** The immunofluorescence results of FTO in ipsilateral (Ipsi) and contralateral (Con) ACC 28 d after CCI (*n* = 4, **P < 0.01). **(D)** The IF double staining of FTO (red) and NeuN (a neuronal marker, green), GFAP (an astrocyte marker, green), or ox42 (a microglia marker, green) in contralateral ACC 28 days after CCI. Scale bar, 100 μm. FTO, fat mass and obesity-related protein.

To detect the role of FTO in anxiety- and depression-like behaviors caused by NP in ACC, the changes in FTO mRNA and protein expression after anxiety- and depression-like behaviors after surgery were first analyzed using qRT-PCR and Western blot. The results showed that the levels of FTO mRNA and proteins were significantly downregulated 7 days after CCI and maintained until 28 days after CCI (Figures 2A,B). To further confirm the above results, the expression profile of FTO in ACC was analyzed using the IF technique. FTO was widely expressed in ACC, and FTO immunoreactivity was decreased in the contralateral side, compared with that in the ipsilateral side of ACC 28 days after CCI (Figure 2C). The above evidence suggest that NP can lead to the decreased FTO expression in ACC. IF results showed that FTO was highly co-expressed with neuronal nuclear antigen (NeuN) and sparsely expressed with glial fibrillary acidic protein (GFAP) or OX42 (Figure 2D).

### Overexpression of Fat Mass and Obesity-Related Protein in the Anterior Cingulate Cortex of CCI Mice Reversed the Anxiety- and Depression-Like Behaviors Induced by Neuropathic Pain

**FIGURE 3:**
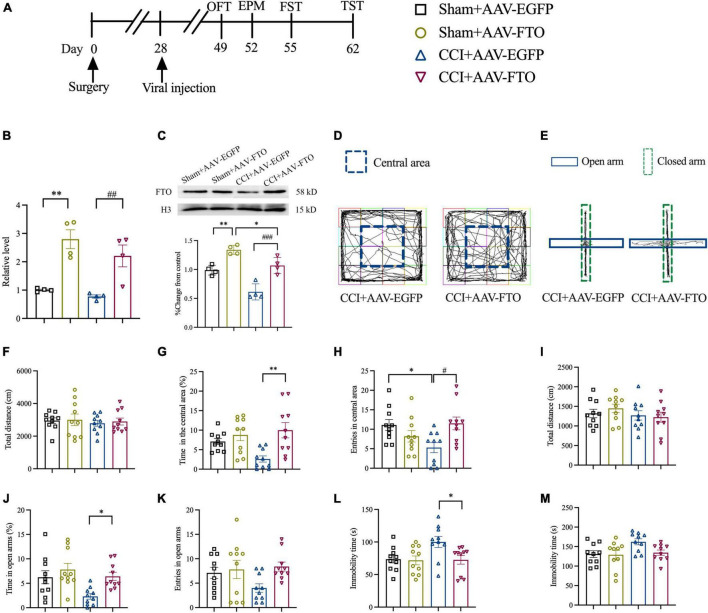
Overexpression of FTO in ACC reversed anxiety- and depression-like behaviors in CCI mice. **(A)** The schematic diagram of the experimental arrangement. **(B,C)** qRT-PCR and Western blot results showed that the expression levels of FTO mRNA and protein in mice ACC 21 days after AAV injection (*P < 0.05, **P < 0.01 vs. Sham-AAV-EGFP; *^##^P* < 0.01, *^###^P* < 0.001 vs. CCI-AAV-EGFP, *n* = 4). **(D)** OFT trajectory. **(E)** EPM trajectory. **(F)** The total movement distance of mice during the OFT. **(G)** The proportion of residence time in the central area (**P < 0.01 vs. CCI-AAV-EGFP). **(H)** The number of entries of the central area (*P < 0.05 vs. Sham-AAV-EGFP; *^#^P* < 0.05 vs. CCI-AAV-EGFP). **(I)** The total movement distance of mice during the EPM. **(J)** The proportion of exploration time in open arm (*P < 0.05 vs. CCI-AAV-EGFP). **(K)** The number of entries of the open arm. **(L,M)** The immobility time during the FST and TST (*P < 0.05 vs. CCI-AAV-EGFP). *n* = 10.

To explore the function of FTO in ACC and the anxiety- and depression-like behaviors induced by NP, recombinant AAV-FTO overexpressing FTO was synthesized. AAV was injected into contralateral ACC by stereotactic injection at 28 days postoperatively (Figure 3A). Twenty-one days after the AAV injection, AAV-FTO was mainly expressed in ACC neurons and minimally in astrocytes and microglia (Supplementary Figures 1A–D). The qRT-PCR and Western blot results showed that the mRNA and protein levels of FTO were significantly upregulated in the Sham group and CCI group 21 days after injection of AAV-FTO, compared with injection with AAV-EGFP (Figures 3B,C). The results of OFT showed that the percentage of time spent in the central area and entries of the central area in the CCI group were significantly induced after overexpression of FTO after injection with AAV-FTO, compared with injection with AAV-EGFP. However, no significant difference in the total movement distance was observed (Figures 3D,F–H). EPM results showed that, CCI mice with overexpression of FTO had a significantly increased percentage of time in the open arms. The entries of the open arms had a tendency to shorten, compared with CCI mice injected with AAV-EGFP, but the tendency was not statistically significant. In addition, there is no significant difference in the total movement distance was observed (Figures 3E,I–K). The results of FST showed that the immobility time of CCI mice with overexpression of FTO were significantly shorter than that of CCI mice injected with AAV-EGFP (Figure 3L). The results of TST showed that the immobility time of CCI mice with overexpression of FTO have a tendency to shorten than that of CCI mice injected with AAV-EGFP, but the tendency was not statistically significant (Figure 3M). These results show that reversing the downregulation of FTO in ACC of CCI mice could ameliorate NP-induced anxiety- and depression-like behaviors.

### Knockdown of Fat Mass and Obesity-Related Protein in the Anterior Cingulate Cortex of Naïve Mice Induced Anxiety- and Depression-Like Behaviors

**FIGURE 4:**
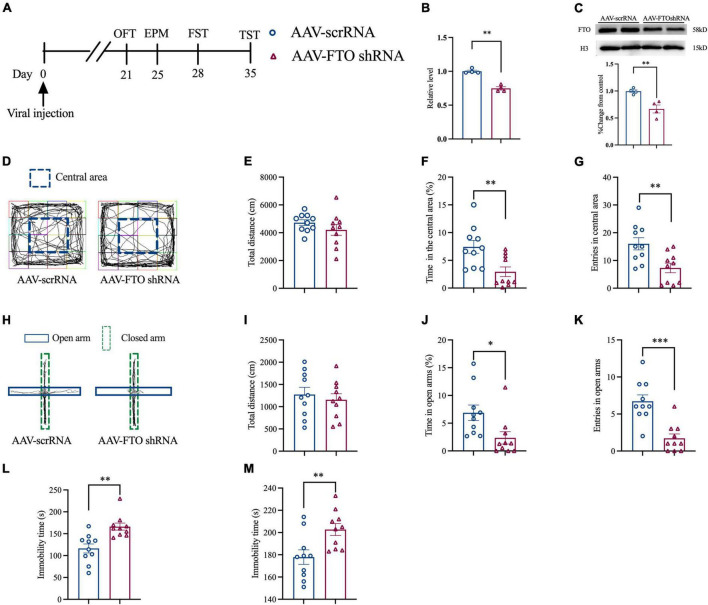
Knockdown FTO in ACC induced anxiety- and depression-like behaviors in naive mice. **(A)** The schematic diagram of the experimental arrangement. **(B,C)** qRT-PCR and Western blot results of the expression of FTO mRNA and protein in naive mice 21 days after transfection with AAV (**P < 0.01, ***P < 0.001 vs. AAV- scrRNA). **(D)** OFT trajectory. **(E)** total movement distance during OFT. **(F)** Percentage of residence time in the central area during OFT (**P < 0.01 vs. AAV- scrRNA). **(G)** The number of entering the central area during OFT (**P < 0.01 vs. AAV- scrRNA). **(H)** EPM trajectory. **(I)** Total movement distance during EPM. **(J)** Percentage of exploration time in open arm during EPM (*P < 0.05 vs. AAV- scrRNA). **(K)** The number of mice entering the open arm during EPM (***P < 0.001 vs. AAV- scrRNA). **(L,M)** Immobility time of FST and TST (**P < 0.01 vs. AAV- scrRNA).

Thereafter, we explored whether FTO knockdown in ACC could induce anxiety- and depression-like behaviors. Naïve mice were injected with AAV for 21 days, and molecular and behavioral experiments were conducted (Figure 4A). The qRT-PCR and Western blot results showed that, after AAV FTO shRNA was injected into mice ACC, the level of FTO mRNA and protein were significantly decreased (Figures 4B,C). OFT results showed that after ACC FTO knockdown, the percentage of time spent in the central area and entries of the central area in the CCI group were significantly decreased. However, no significant difference in total movement distance was observed (Figures 4D–H). EPM results showed that the percentage of time in the open arms and entries of the open arms were significantly reduced, and the total movement distance was also reduced (Figures 4H–K). These findings indicate that, after FTO knockdown, naïve mice generated anxiety-like behavior. FST and TST showed that the resting time of mice was significantly prolonged after ACC FTO knockdown (Figures 4L,M), suggesting that FTO knockdown induced depression-like behaviors in naïve mice.

### Fat Mass and Obesity-Related Protein Affects the Maturation of Brain-Derived Neurotrophic Factor by Regulating Matrix Metalloproteinase-9

**FIGURE 5:**
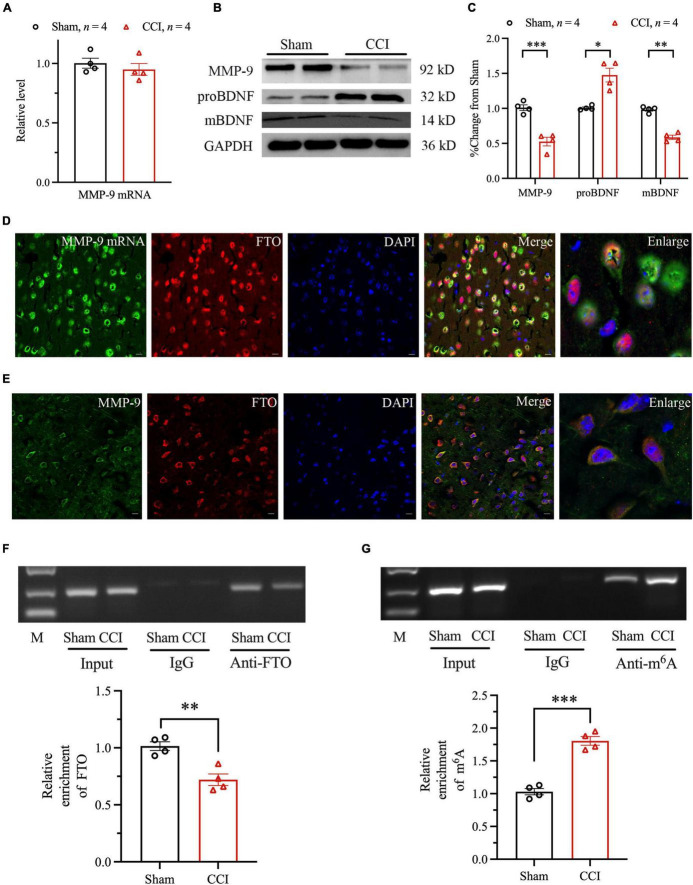
The mechanism of FTO affecting the expression of MMP-9. **(A)** qRT-PCR results of the expression of MMP-9 mRNA in ACC of Sham and CCI mice. **(B,C)** Western blot results of the expression of MMP-9, proBDNF, and mBDNF (*P < 0.05, **P < 0.01, ***P < 0.001 vs. Sham). **(D)** The representative images of the original hybridization of MMP-9 mRNA and co-labeling of FTO immunofluorescence in ACC. **(E)** The cellular expression patterns of FTO protein and MMP-9 protein. **(F)** RIP results of FTO and MMP-9 mRNA in ACC after CCI in mice (**P < 0.01 vs. Sham). **(G)** RIP results of m^6^A and MMP-9 mRNA in ACC after CCI in mice (***P < 0.001 vs. Sham).

Furthermore, the RNA posttranscriptional modifications regulated by FTO were examined. Increasing evidence suggest that MMPs may be involved in the pathogenesis of depression and anxiety. The expression level of MMP-2 and MMP-9 protein level in the brain and serum of patients with recurrent depression was lower than that of healthy people (Bobinska et al., 2016). BDNF is one of the most widely distributed and studied neurotrophic factors in the mammalian brain. It is an important part of normal brain function, including nerve cell survival, learning and memory, and synaptic plasticity (Arancio and Chao, 2007). The mBDNF and proBDNF play different roles in CNS. Several studies have shown that the conversion or deficiency of proBDNF to mBDNF may damage the plasticity of neurons, resulting in anxiety- and depression-like behaviors. Studies have shown that MMP-9 participates in anxiety- and depression-like behaviors in mouse hippocampus in a BDNF-dependent manner. Our current research found that the MMP-2 mRNA and protein level did not change significantly (Supplementary Figures 2A,B). MMP-9 mRNA level did not change significantly 28 days after CCI (Figure 5A). However, Western blot analysis revealed that MMP-9 protein level was significantly downregulated, compared with Sham group (Figures 5B,C). We further explored whether FTO downregulation in ACC regulates anxiety- and depression-like behaviors through MMP-9/BDNF axis coordination in NP model. Our research has found that the protein levels of proBDNF and mBDNF were significantly upregulated and downregulated, respectively 28 days after CCI (Figures 5B,C). To prove the way by which FTO regulates MMP-9, the distribution patterns of FTO and MMP-9 mRNA were investigated by combining ISH with IF. The results showed that FTO and MMP-9 mRNA were highly co-labeled in ACC neurons (Figure 5D). Thereafter, IF double staining showed that FTO and MMP-9 were co-expressed in ACC neurons (Figure 5E). In addition, the RIP experiment further showed that FTO could bind to ACC MMP-9 mRNA, and the binding of FTO to MMP-9 mRNA decreased after CCI (Figure 5F). The results of m^6^A RIP showed that m^6^A modification of MMP-9 mRNA in mice ACC increased after CCI (Figure 5G). Therefore, the downregulation of MMP-9 protein level after CCI may be due to changes in m^6^A modification level in mRNA.

**FIGURE 6:**
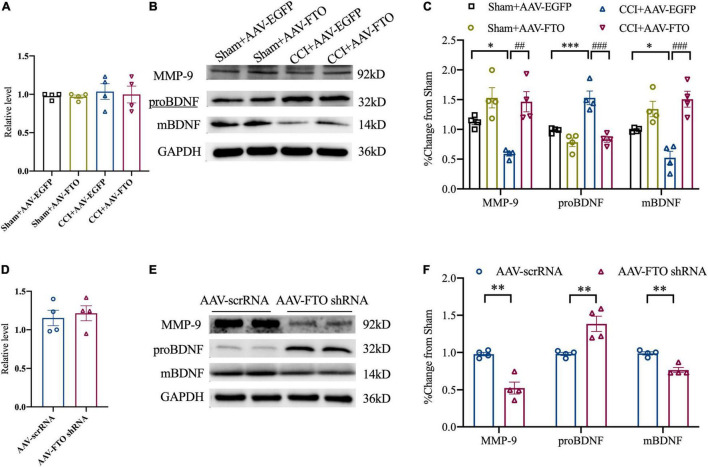
Fat mass and obesity-related protein affects the maturation of brain-derived neurotrophic factor by regulating MMP-9. **(A)** The expression of MMP-9 mRNA 21 days after ACC injection of AAV in Sham and CCI mice. **(B,C)** The expression of MMP-9, proBDNF, and mBDNF proteins 21 days after ACC injection of AAV-FTO in the Sham group and CCI group (*P < 0.05, ***P < 0.001 vs. Sham-AAV-EGFP, *^##^P* < 0.01, *^###^P* < 0.001 vs. CCI-AAV-FTO). **(D)** The expression of MMP-9 mRNA 21 days after ACC injection of AAV in naïve mice. **(E,F)** Changes in protein levels of MMP-9, proBDNF, and mBDNF in naïve mice after ACC injection of AAV for 21 days (**P < 0.01 vs. AAV scrRNA).

The qRT-PCR analysis revealed that MMP-9 mRNA level in ACC of CCI mice did not change significantly. However, Western blot analysis showed that, compared with Sham and CCI mice injected with AAV EGFP, Sham and CCI mice with FTO overexpression in ACC had significantly overexpressed MMP-9 protein levels. Accordingly, the protein levels of proBDNF and mBDNF were downregulated and upregulated, respectively (Figures 6A–C). After knockdown of FTO expression in ACC of naïve mice, the mRNA level did not change significantly, but the MMP-9 protein level was significantly downregulated. Additionally, the increase in proBDNF protein levels and decrease in mBDNF protein levels in ACC after CCI could be simulated (Figures 6D–F). These results show that the downregulation of FTO in ACC of NP mice affected the balance of proBDNF/mBDNF in an MMP-9-dependent manner, and finally led to anxiety- and depression-like behaviors.

## Discussion

About 9% of the world’s population suffer from NP (van Hecke et al., 2014). NP often affect patients’ quality of life in many aspects, such as emotional, cognitive, and sleep disorders (Radat et al., 2013). Anxiety is one of the common emotional disorders in patients with chronic pain, with an incidence of 27% in patients with NP (Kremer et al., 2021). Several studies have explored the close relationship between pain and depression. In patients with chronic pain, the average prevalence of severe depression is between 18 and 85%, whereas in patients with depression, the incidence of chronic pain is 51.8–59.1% (von Knorring et al., 1983; Lee et al., 2009; Aguera-Ortiz et al., 2011). Our study found that the PWT and PWL of mice decreased significantly after CCI, and lasted up to 28 days. OFT and EPM showed that anxiety behaviors appeared in CCI mice after operation. FST and TST showed that the immobility time of mice was significantly prolonged in CCI mice, suggesting that NP can lead to depression-like behaviors in mice. These results show that NP can induce anxiety- and depression-like behaviors in mice.

In recent years, epigenetic modification at the transcriptional level has advanced in the occurrence of NP, but epigenetic regulation at the translation level remains incomplete (Randon et al., 2020). Recently, epigenetic molecules, such as brain m^6^A/M, play an important role in regulating the expression of genes related to stress depression. RNA m^6^A modification is prepared by RNA demethylases, such as FTO (Jia et al., 2011) and ALKB homolog 5 (ALKBH5) (Zheng et al., 2013), and RNA methyltransferase complexes, such as methyltransferase-like 3 (METTL3), METTL14 (Liu et al., 2014), and WTAP (Ping et al., 2014). After peripheral nerve injury, FTO in DRG is involved in the generation and maintenance of hyperalgesia in mice (Li et al., 2020) and participates in hemorrhage-induced thalamic pain (Fu et al., 2021). Several studies have shown that FTO plays an important role in many biological processes, including control of neurogenesis (Li et al., 2017), regulation of memory formation, and emotional changes (Spychala and Ruther, 2019). In Rivera et al. (2012) reported that the interaction among FTO gene, depression, and BMI suggested that FTO is involved in the mechanism of association between depression and obesity. Another study found that FTO deficiency increased anxiety and impaired working memory in hippocampus (Spychala and Ruther, 2019). Our study found that the expression of FTO mRNA and protein in mouse ACC had decreased significantly 7 days after CCI and could last until 28 days after CCI. The results of IF double staining showed that FTO in ACC was mainly expressed in neurons and minimally in astrocytes and microglia, which was consistent with the results of a previous study (Fu et al., 2021). Our study showed that FTO was significantly down-regulated in the contralateral ACC of CCI mice compared with the Surgery on the ipsilateral ACC. Kuei-Sen Hsu et al’s research showed that excitatory neurons in the ACC send direct descending projections to the contralateral dorsal horn of the lumbar spinal cord *via* the dorsal corticospinal tract (Chiou et al., 2016). This lateralization of ACC in pain is consistent with our study. However, Guang Yang et al’s research suggested that the activation of ACC neurons occurred bilaterally upon noxious stimulation to either contralateral or ipsilateral in hind paws (Zhao et al., 2018). It is suggested that there may be differences in the laterality of changes in different proteins in ACC. Our study provided the evidence that FTO was decreased in mice with anxiety- and depression-like behaviors induced by NP, which was reversed by overexpression of FTO in ACC. Contrarily, FTO knockdown in ACC of naïve mice can induce anxiety- and depression-like behaviors in mice. However, conflicting evidence found that FTO global knockout mice were more resistant to stress stimulation and reduced anxiety- and depression-like behaviors by alterations of gut microbiota (Sun et al., 2019). The conclusions about the association between FTO and depression may be different when considering the heterogeneity of depression.

Matrix metalloproteinases has a wide range of substrate specificity and strictly regulates tissue specificity, which can be by remodeling of extracellular matrix (ECM). Furthermore, degradation of various proteins plays an important role in cell proliferation, migration, differentiation, apoptosis, angiogenesis, tissue repair, immune response, and inflammatory response (Itoh, 2015; Kessenbrock et al., 2015; Chang et al., 2016). Studies have shown that MMP-2 and MMP-9 play key roles in the occurrence and development of chronic pain by activating microglia in the DRG and spinal cord (Kawasaki et al., 2008) and participates in the regulation of oxidative stress and inflammatory response through a variety of ways (Fingleton, 2017). Regarding mental diseases, MMP-2 have a role in the pathophysiology of MDD (Omori et al., 2020). However, our experimental results showed that MMP-2 level did not change significantly. Therefore, we focused on MMP-9 in the later study. MMP-9 enhances nervous system excitation/inhibition balance and neuronal population dynamics, which are very important for the normal maintenance of emotion and cognition (Alaiyed et al., 2020). Furthermore, MMP-9 may be involved in the growth of dendrites and dendritic spines of vertebral neurons and plays an important role in the formation of learning and memory (Smith et al., 2014). The increased anxiety in FTO^–/–^ mice could be caused by the restricted neuronal differentiation in the hippocampus induced by reduced level of MMP-9 (Spychala and Ruther, 2019), and the mBDNF level is reduced by loss of FTO in the hippocampus, but the mechanism by which FTO regulates the amount of MMP-9 cannot be clearly determined. Our study found that, with the downregulation of FTO, the level of MMP-9 protein in ACC was significantly downregulated, but no significant change was observed in its mRNA level in NP mice. RNA methylation sites on the MMP-9 mRNA could be predicted. One study revealed that m^6^A methylation is suspected to take place on each of the 13 MMP-9 exons in mice (Liu et al., 2015). The RIP experiments on FTO and MMP-9 mRNA showed that the binding of FTO to MMP-9 mRNA decreased after CCI, whereas those on m^6^A and MMP-9 mRNA showed that m^6^A modification on MMP-9 mRNA increased. These results suggest that, as a demethylase, FTO regulates the expression of MMP-9 through epigenetic modification. The decrease in MMP-9 protein level may be caused by the increase in m^6^A modification on MMP-9 mRNA mediated by FTO downregulation. In addition, the primers of MMP-9 mRNA are located on the exon 2 of MMP-9 mRNA according to the relevant information provided by NCBI.

Brain-derived neurotrophic factor belongs to the neurotrophic protein family and is widely expressed in the central nervous system. It is mainly synthesized by nerve cells and astrocytes (Thomas et al., 2016). It is an important part of normal brain function, including nerve cell survival, learning and memory, and synaptic plasticity (Arancio and Chao, 2007). BDNF plays an important role in the pathophysiological process of depression and anxiety. According to previous studies, FTO can affect neurogenesis, and the loss of FTO may lead to the change in the BDNF signaling pathway in the hippocampus (Li et al., 2017), resulting in the increase in anxiety and the loss of working memory in mice. BDNF levels were significantly reduced in the brain and serum of patients with anxiety and depression (Wang et al., 2015; Castren and Kojima, 2017). Its precursor, preproBDNF, is synthesized in the endoplasmic reticulum and then processed into the precursor protein, proBDNF. ProBDNF can be further processed into mBDNF. Studies have shown that proBDNF/mBDNF imbalance may lead to anxiety or depression (Lin et al., 2019). Our study found that, after the downregulation of FTO in ACC, the transformation from proBDNF to mBDNF was blocked, the level of proBDNF protein increased, and the expression of mBDNF protein decreased, which was reversed by overexpression of FTO in ACC. Knockdown of FTO in ACC of control mice can simulate the increase in proBDNF protein level and the decrease in mBDNF protein expression in ACC of CCI mice, and can induce anxiety- and depression-like behaviors in mice. According to previous studies, MMP-9 is involved in the extracellular processing of transformation from proBDNF to mBDNF (Pang et al., 2004; Rivera et al., 2017). Combined with our experimental results, the FTO in ACC of NP mice reduces the protein expression of MMP-9 by affecting the translation efficiency of MMP-9 mRNA, thus hindering the transformation from proBDNF to mBDNF and resulting in anxiety- and depression-like behaviors in mice.

Studies have shown that ACC neurons may receive nociceptive information from the thalamus and somatosensory cortex and emotional information of fear/anxiety from the amygdala. This feature allows ACC neurons to integrate sensory input on the one hand and anxiety signals on the other. Additionally, clinical research have shown that pain stimulation can enhance the metabolism of neurons in ACC, and its degree is related to the intensity of stimulation (Lenz et al., 1998; Wager et al., 2013). ACC has been reported to participate in anxiety- and depression-like behaviors in human and animal studies (Osuch et al., 2000; Etkin et al., 2011; Kim et al., 2011; Mochcovitch et al., 2014; Barthas et al., 2015). These findings highlighted the potential importance of ACC synaptic plasticity in the pathophysiology of depression. Recently, one study revealed that early-life inflammation promotes depressive symptoms in adolescence by microglial engulfment of dendritic spines (Cao et al., 2021). BDNF efficiently modifies synaptic strength and can act as an instructor of synaptic plasticity (Colucci-D’Amato et al., 2020). Another study suggested that chronic stress induced reduction in dendritic spine density and number of branches, which were reversed by overexpression of FTO in the hippocampus (Spychala and Ruther, 2019). Our study found that the FTO in ACC can reduce the expression of mBDNF by regulating MMP-9 in NP. Therefore, the downregulated mBDNF is involved in the occurrence of anxiety- and depression-like behaviors by affecting the prominent plasticity of neurons in ACC. Considering that FTO ensures proper ACC function by regulating BDNF processing through the control of ACC MMP-9 level, we propose FTO as a possible new target for developing novel approaches for the treatment of diseases associated with ACC disorders by regulating the processing of mBDNF.

## Conclusion

Our study revealed the epigenetic mechanism of FTO-triggered MMP-9 downregulation in ACC after CCI. FTO may be an endogenous trigger of NP-induced anxiety- and depression-like behaviors, which will improve our understanding of mechanism of anxiety- and depression-like behaviors induced by NP, and may provide potential targets for developing new therapeutic strategies.

## Data Availability Statement

The original contributions presented in the study are included in the article/Supplementary Material, further inquiries can be directed to the corresponding author/s.

## Ethics Statement

The animal study was reviewed and approved by the Institutional Animal Care and Use Committee of Zhengzhou University.

## Author Contributions

X-LW and XW participated in the study design and performed the stereotaxic surgeries for lentiviral injections, data processing, immunohistochemical procedures, blinded cell counting of FTO and MMP9 labeling, and manuscript drafting. J-JY performed the micro dialysis experiments. Z-YW and NX participated in the western blot experiments and writing of the manuscript. H-WG, C-HL, and W-TW participated in data collection. FX and WZ participated in the study design, discussion of the results, and modified of the manuscript. All authors have read and approved the final version of the manuscript finalized by WZ.

## Conflict of Interest

The authors declare that the research was conducted in the absence of any commercial or financial relationships that could be construed as a potential conflict of interest.

## Publisher’s Note

All claims expressed in this article are solely those of the authors and do not necessarily represent those of their affiliated organizations, or those of the publisher, the editors and the reviewers. Any product that may be evaluated in this article, or claim that may be made by its manufacturer, is not guaranteed or endorsed by the publisher.

## Footnotes

1
https://www.ncbi.nlm.nih.gov/tools/primer-blast
