# Supplementary figures and images for: Downregulation of Fat Mass and Obesity-Related Protein in the Anterior Cingulate Cortex Participates in Anxiety- and Depression-Like Behaviors Induced by Neuropathic Pain
Source: Front Cell Neurosci. 2022 May 12;16:884296. doi: 10.3389/fncel.2022.884296 (PMC9133794; doi:10.3389/fncel.2022.884296)

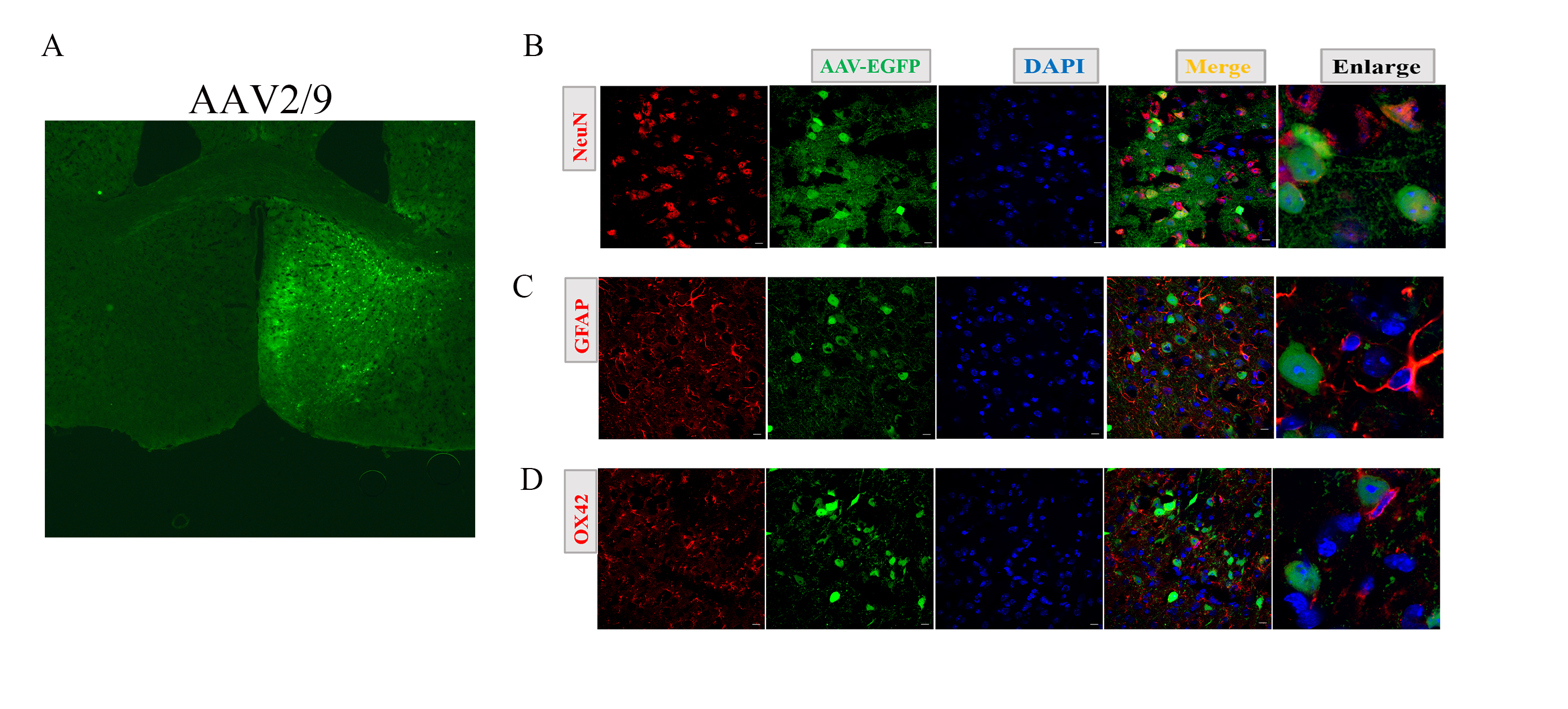

Supplement: Supplementary file 1 [file Image_1.JPEG]

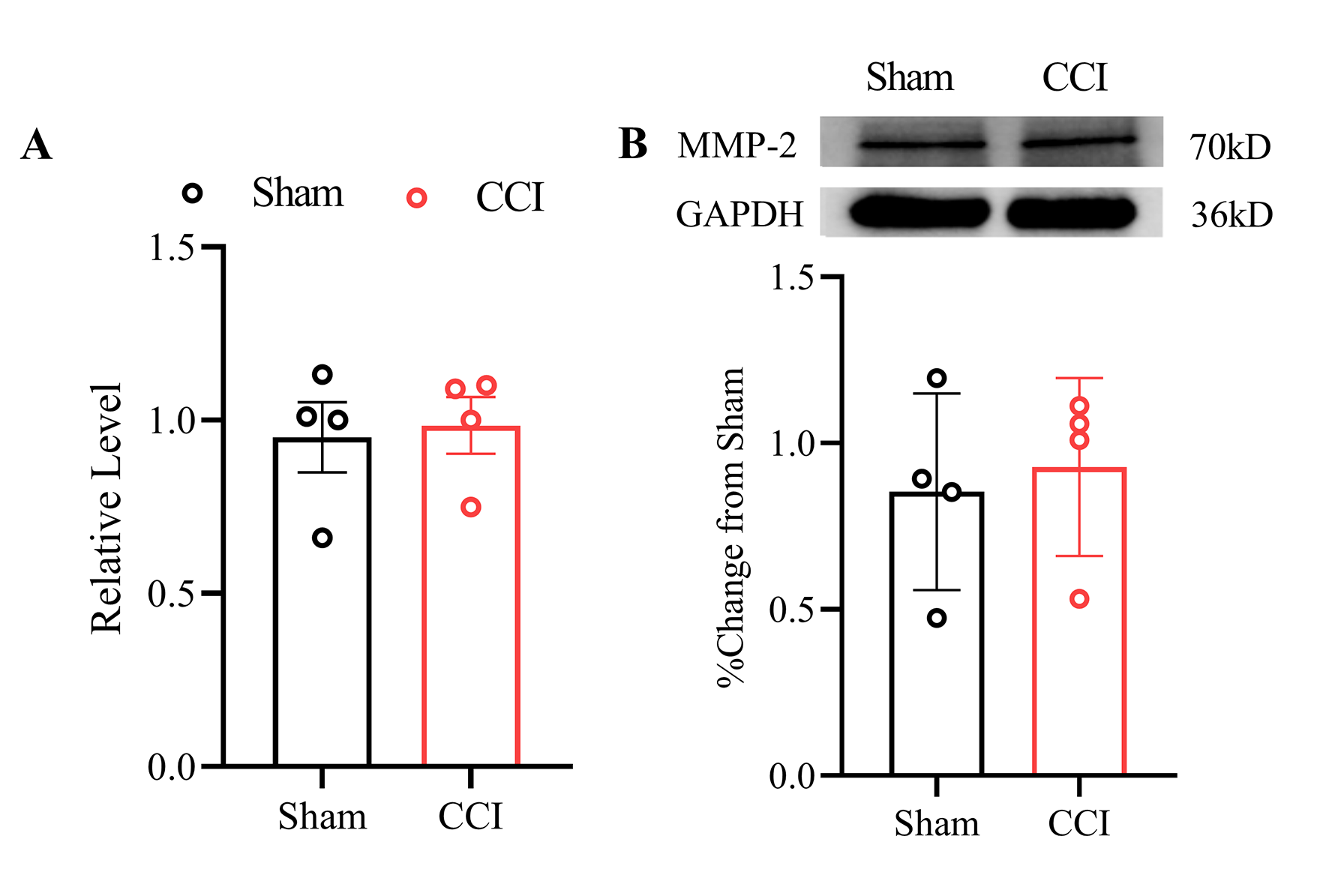

Supplement: Supplementary file 2 [file Image_2.TIF]
